# Phospholipase A_2 _inhibitors protect against prion and Aβ mediated synapse degeneration

**DOI:** 10.1186/1750-1326-5-13

**Published:** 2010-04-08

**Authors:** Clive Bate, Mourad Tayebi, Alun Williams

**Affiliations:** 1 Department of Pathology and Infectious Diseases, Royal Veterinary College, Hawkshead Lane, North Mymms, Herts, AL9 7TA, UK; 2 Department of Veterinary Medicine, University of Cambridge, Madingely Road, Cambridge, CB3 OES, UK

## Abstract

**Background:**

An early event in the neuropathology of prion and Alzheimer's diseases is the loss of synapses and a corresponding reduction in the level of synaptophysin, a pre-synaptic membrane protein essential for neurotransmission. The molecular mechanisms involved in synapse degeneration in these diseases are poorly understood. In this study the process of synapse degeneration was investigated by measuring the synaptophysin content of cultured neurones incubated with the prion derived peptide (PrP82-146) or with Aβ_1-42_, a peptide thought to trigger pathogenesis in Alzheimer's disease. A pharmacological approach was used to screen cell signalling pathways involved in synapse degeneration.

**Results:**

Pre-treatment with phospholipase A_2 _inhibitors (AACOCF_3_, MAFP and aristolochic acids) protected against synapse degeneration in cultured cortical and hippocampal neurones incubated with PrP82-146 or Aβ_1-42_. Synapse degeneration was also observed following the addition of a specific phospholipase A_2 _activating peptide (PLAP) and the addition of PrP82-146 or Aβ_1-42 _activated cytoplasmic phospholipase A_2 _within synapses. Activation of phospholipase A_2 _is the first step in the generation of platelet-activating factor (PAF) and PAF receptor antagonists (ginkgolide B, Hexa-PAF and CV6029) protected against synapse degeneration induced by PrP82-146, Aβ_1-42 _and PLAP. PAF facilitated the production of prostaglandin E_2_, which also caused synapse degeneration and pre-treatment with the prostanoid E receptor antagonist AH13205 protected against PrP82-146, Aβ_1-42 _and PAF induced synapse degeneration.

**Conclusions:**

Our results are consistent with the hypothesis that PrP82-146 and Aβ_1-42_trigger abnormal activation of cytoplasmic phospholipase A_2 _resident within synapses, resulting in elevated levels of PAF and prostaglandin E_2_that cause synapse degeneration. Inhibitors of this pathway that can cross the blood brain barrier may protect against the synapse degeneration seen during Alzheimer's or prion diseases.

## Background

In the transmissible spongiform encephalopathies, otherwise known as the prion diseases, changes in synaptic function and a reduction in synaptophysin levels within the brain occur at a time before any gross neuronal loss is observed [1-3]. These synaptic alterations are associated with the accumulation of a differentially folded, and protease-resistant isoform (PrP^Sc^), of the host encoded cellular prion protein (PrP^C^) [4]. The formation of PrP^Sc ^is accompanied by a decreased expression of proteins involved in exocytosis and neurotransmission, such as synaptophysin, SNAP-25 and synapsins in the brains of scrapie-infected mice [2,5] and in humans affected with Creutzfeldt-Jakob disease (CJD) [6].

The molecular mechanisms that underlie synapse degeneration in prion diseases are not understood. Such processes have been examined by incubating cultured neurones with PrP^Sc ^or specific prion-derived peptides. A major PrP fragment spanning amino acid residues 81-82 to 144-153 was isolated from the brains of patients with the hereditary prion disease Gerstmann-Sträussler-Scheinker disease [7]. Synthetic peptides containing amino acid residues 82 to 146 (PrP82-146) had similar structural and biochemical properties to PrP^Sc ^suggesting that this fragment was the neurotoxic species generated in prion diseases. This hypothesis was strengthened by observations that both partially purified PrP^Sc ^preparations and PrP82-146 caused synapse degeneration in cortical and hippocampal neurones [8]. The effect of PrP82-146 on synapses in neuronal cultures was measured using an enzyme linked immunoassay (ELISA) to quantify the amount of synaptophysin [9]. Synaptophysin is a pre-synaptic membrane protein essential for neurotransmitter release and the recycling of synaptic vesicles and hence neurotransmission [10-13]. The amount of synaptophysin has been used to access synaptic density in the brain [14,15] and in cultured neurones [8]. Although immunocytochemistry is commonly used to examine synapse density this method is susceptible to errors in counting and field selection. The use of an ELISA overcame such problems by measuring synaptic density throughout neuronal cultures.

Synaptic failure is also thought to contribute to the neuropathogenesis of Alzheimer's disease (AD) [16] and the loss of synaptic proteins is the best correlate of dementia in AD [14,17-20]. The amyloid hypothesis of AD pathogenesis maintains that the primary event is the production of neurotoxic amyloid-β (Aβ) peptides following the proetolytic cleavage of the amyloid precursor protein into different fragments [21,22]. These fragments include Aβ_1-42 _which is widely regarded as the main pathogenic species in AD. Recent studies showed the importance of small soluble oligomers of Aβ or Aβ derived diffusible ligands in neurotoxicity [23,24]. In this study we sought to determine whether PrP82-146 and Aβ induced synapse degeneration was mediated through specific cell signalling pathways. We report that PrP82-146 and Aβ_1-42 _induced synapse degeneration was prevented by pharmacological inhibition of PLA_2 _and that both PrP82-146 and Aβ_1-42 _peptides increased activation of cytoplasmic phospholipase A_2 _(cPLA_2_) within synapses suggesting that activation of this enzyme triggers synapse degeneration. This hypothesis was supported by the observation that the synapse degeneration was also observed following the addition of a specific PLA_2 _activating peptide (PLAP). Activation of PLA_2 _is the first step in the production of bioactive prostaglandins and platelet-activating factor (PAF), specific antagonists of which also reduced PrP82-146 and Aβ_1-42 _induced synapse degeneration.

## Results

### PLA_2 _inhibitors protected against PrP82-146 induced synapse degeneration

**Figure 1:**
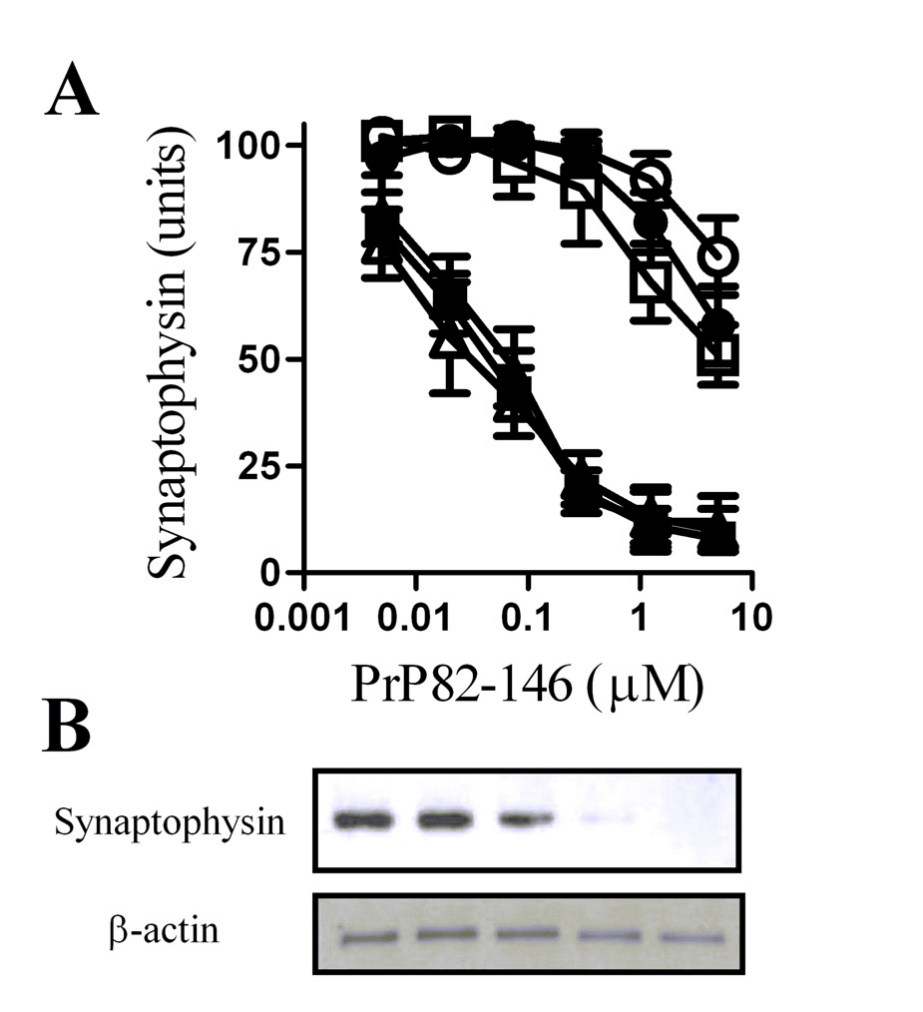
**PLA_2 _inhibitors protected cortical neurones against PrP82-146 induced synapse degeneration**. (A) The synaptophysin content of cortical neurones pre-treated for with a vehicle control (■), 1 μM AACOCF_3 _(○), 1 μM MAFP (●), 5 μg/ml aristolochic acid (□), 10 μM U73122 (▲) or 10 μM ethyl-18-OCH_3 _(△) and subsequently incubated with varying concentrations of PrP82-146 for 24 hours. Values shown are the mean average amount of synaptophysin ± standard deviation (SD), n = 15. (B) Immunoblots showing the amount of synaptophysin and β-actin in cell extracts from neurones incubated with PrP82-146 (1.25 - 0.05 μM).

The addition of the prion derived peptide PrP82-146 reduced the synaptophysin content of cortical neurones indicative of a loss of synapses. This effect was a consequence of the specific amino acid sequence of PrP82-146 as a control peptide (PrP82-146scram) did not affect synapses [8]. The synaptophysin content was reduced by greater than 80% by 1 μM PrP82-146 (Figure 1A) which did not affect neuronal survival as measured by thiazyl blue tetrazolium (98% neuronal survival ± 6 compared with 100% ± 6, n = 9, P = 0.53). Immunoblot analysis showed that PrP82-146 caused a dose-related reduction in the amounts of synaptophysin in cell extracts without affecting the amounts of β-actin (Figure 1B).

To determine if the degeneration of synapses resulted from activation of specific cell signalling pathways cortical neurones were pre-treated with inhibitors of some common cell signalling pathways and incubated with PrP82-146. Pre-treatment with the PLA_2 _inhibitors (1 μM AACOCF_3_, 1 μM MAFP or 5 μg/ml aristolochic acids) protected against the PrP82-146 mediated decrease in synaptophysin (Figure 1A). The concentration of PrP82-146 required to reduce the synaptophysin content of vehicle treated cortical neurones by 50% (EC_50_) was approximately 60 nM. By comparison, the EC_50 _of PrP82-146 for cortical neurones treated with 1 μM AACOCF_3 _or 1 μM MAFP was greater than 10 μM, and in neurones pre-treated with 5 μg/ml aristolochic acids the EC_50 _was 5 μM. In contrast, pre-treatment with phospholipase C inhibitors (10 μM U73122 or ethyl-18-OCH_3_) did not affect the PrP82-146 induced loss of synaptophysin from cortical neurones. The synaptophysin content of neurones was not significantly affected by treatment with 5 μM AACOCF_3 _(100 units synaptophysin ± 4 compared to 101 ± 4, n = 12, P = 0.5), 5 μM MAFP (100 ± 4 v 102 ± 6, n = 12, P = 0.4) or 5 μg/ml aristolochic acids (100 ± 4 compared to 98 ± 4, n = 12, P = 0.2) alone showing that these drugs did not stimulate synaptogenesis, nor did they damage synapses.

### PLA_2 _inhibitors protected hippocampal neurones against PrP82-146 induced synapse degeneration

**Figure 2:**
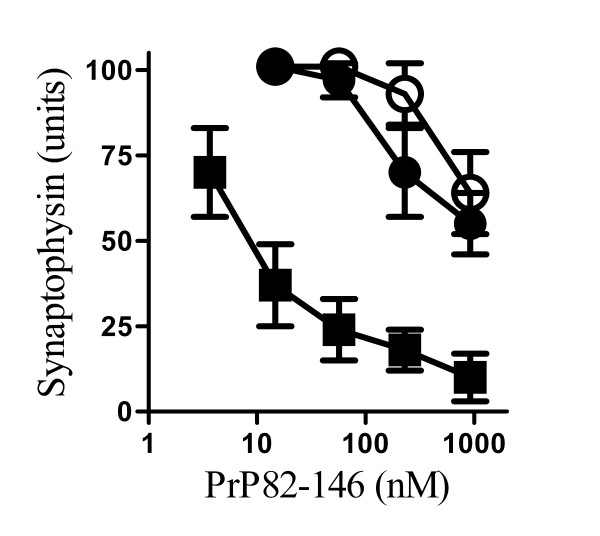
**PLA_2 _inhibitors protected hippocampal neurones against PrP82-146 induced synapse degeneration**. The synaptophysin content of cultured hippocampal neurones pre-treated with a vehicle control (■), 1 μM AACOCF_3 _(○) or 1 μM MAFP (●) and incubated with varying concentrations of PrP82-146 for 24 hours. Values shown are the mean average amount of synaptophysin ± SD, n = 9.

To determine if the effect of PLA_2 _inhibitors was selective for cortical neurones, their effect on hippocampal neurones was also examined. PrP82-146 reduced the amount of synaptophysin in hippocampal neurones; the EC_50 _of PrP82-146 in hippocampal neurones was 10 nM and while the addition of 200 nM PrP82-146 reduced the synaptophysin content to 20% of control cultures, it did not alter cell survival as measured by thiazyl blue tetrazolium (97% cell survival ± 7 compared with 100% ± 9, n = 9, P = 0.7). Pre-treatment with 1 μM AACOCF_3 _or 1 μM MAFP prevented the PrP82-146 mediated decrease in the synaptophysin content of hippocampal neurones and in treated neurones the EC_50 _was increased to greater than 1 μM (Figure 2).

### PLA_2 _inhibitors protected against synapse degeneration induced by Aβ_1-42_

**Figure 3:**
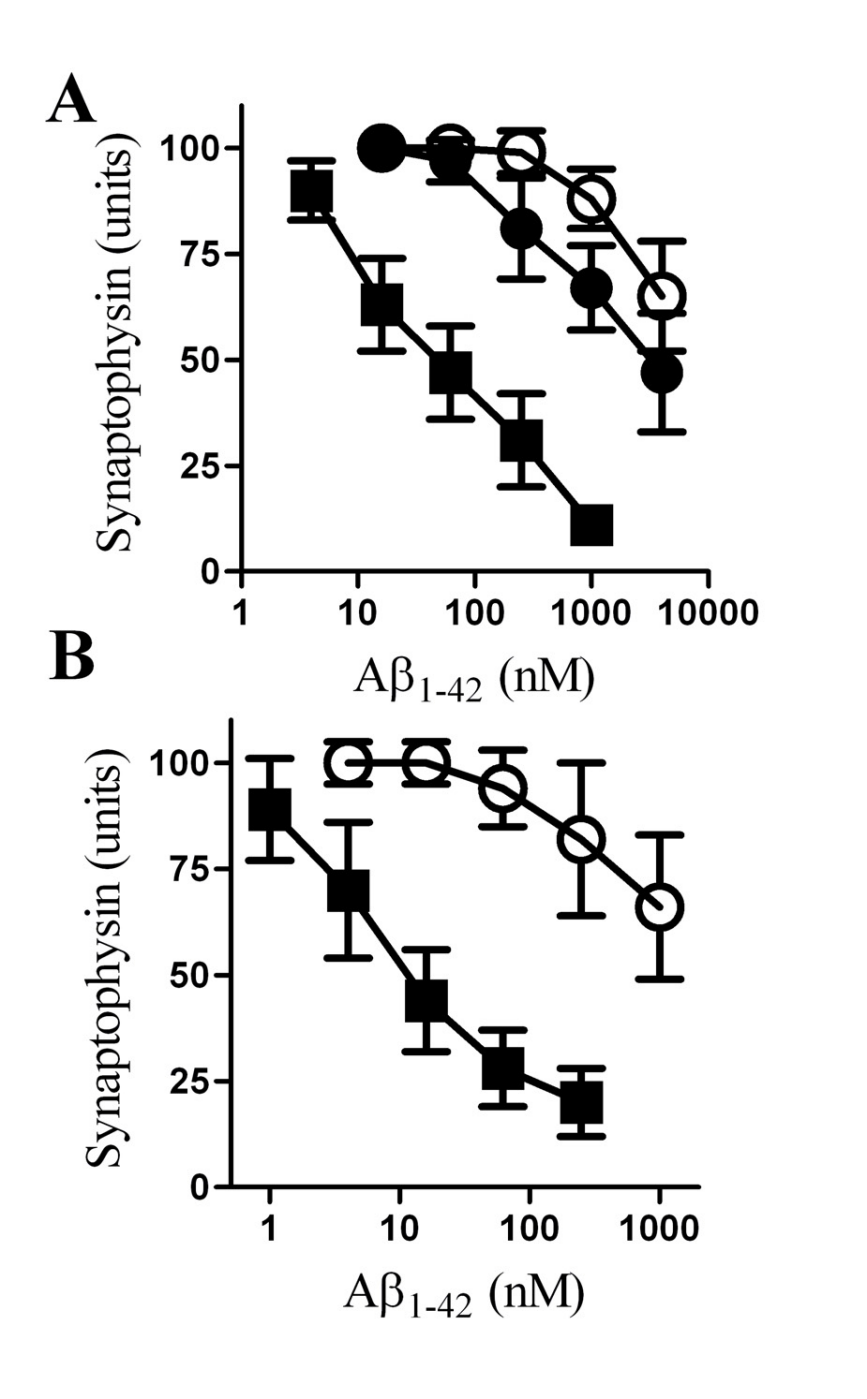
**PLA_2 _inhibitors protected against Aβ_1-42 _induced synapse degeneration**. (A) The synaptophysin content of cortical neurones pre-treated with a vehicle control (■), 1 μM AACOCF_3 _(○) or 1 μM MAFP (●) and incubated with varying concentrations of Aβ_1-42 _for 24 hours. Values shown are the mean average amount of synaptophysin ± SD, n = 12. (B) The synaptophysin content of hippocampal neurones pre-treated with a vehicle control (■) or 1 μM AACOCF_3 _(○) and incubated with varying concentrations of Aβ_1-42 _for 24 hours. Values shown are the mean average amount of synaptophysin ± SD, n = 6.

The synapse degeneration that occurs in AD is thought to be caused by Aβ_1-42 _[25,26]. This process can be modelled *in vitro *and the addition of Aβ_1-42_, but not Aβ_42-1_, reduced the synaptophysin content of cultured cortical and hippocampal neurones [27]. Here we report that pre-treatment with 1 μM AACOCF_3 _or 1 μM MAFP protected cortical neurones against the Aβ_1-42 _induced reduction in synaptophysin content (Figure 3A). While the EC_50 _of Aβ_1-42 _in vehicle treated cortical neurones was 50 nM, the EC_50 _of Aβ_1-42 _for cortical neurones treated with 1 μM AACOCF_3 _was greater than 10 μM. PLA_2 _inhibitors also protected hippocampal neurones against Aβ_1-42 _induced synapse degeneration. Thus, the EC_50 _of Aβ_1-42 _for vehicle treated hippocampal neurones was 10 nM, while in hippocampal neurones pre-treated with 1 μM AACOCF_3 _the EC_50 _of Aβ_1-42 _was increased to greater than 1 μM (Figure 3B).

**Figure 4:**
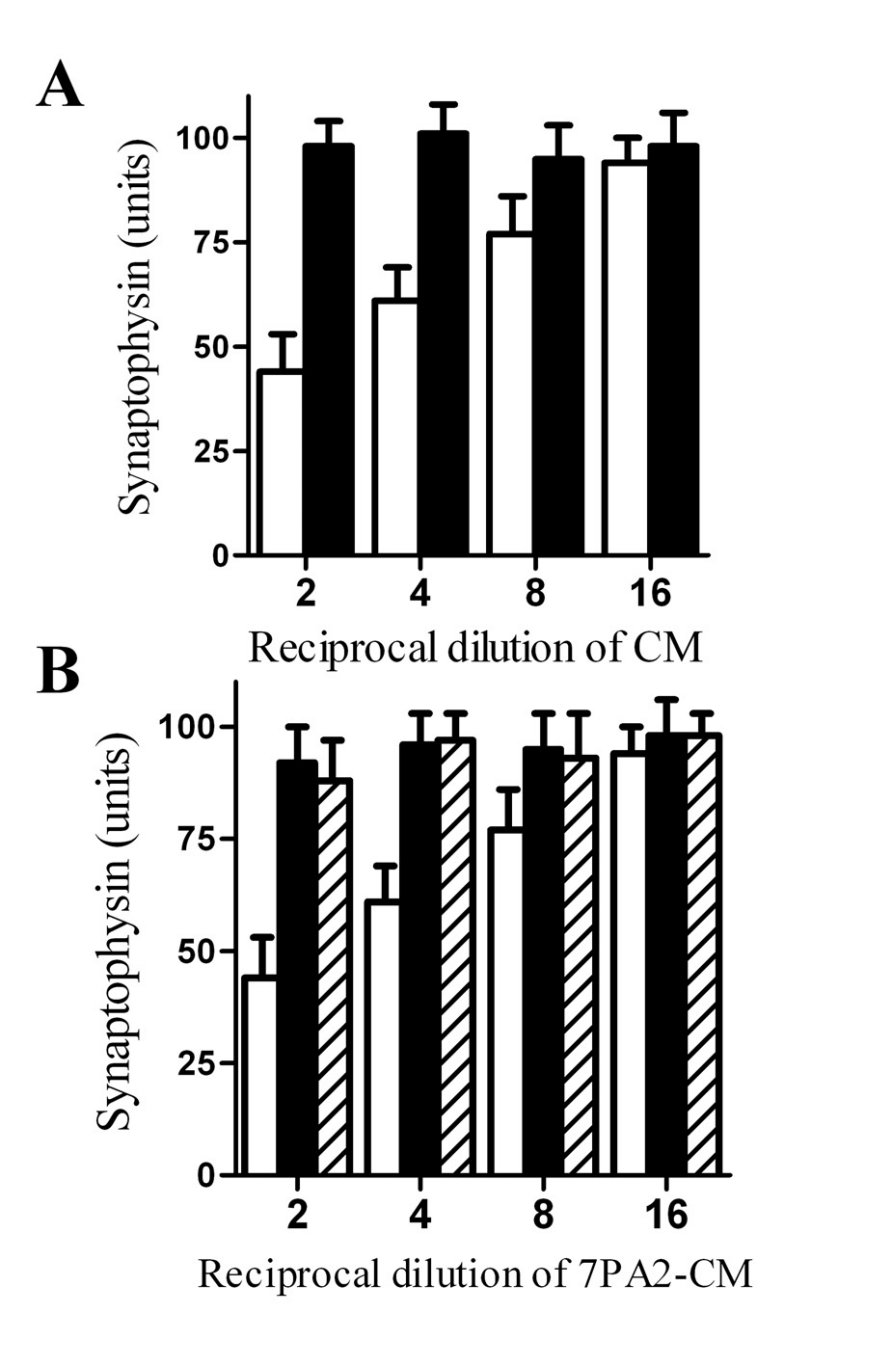
**PLA_2 _inhibitors protected against 7PA2-CM induced synapse degeneration**. (A) The synaptophysin content of cortical neurones incubated for 24 hours with 7PA2-CM (□) or CHO-CM (■). Values shown are the mean average amount of synaptophysin ± SD, n = 12. (B) The synaptophysin content of cortical neurones incubated for pre-treated with a vehicle control (□), 1 μM AACOCF_3 _(■) or 1 μM MAFP (striped bars) and incubated with doubling dilutions of 7PA2-CM for 24 hours. Values shown are the mean average amount of synaptophysin ± SD, n = 12.

Conditioned medium from 7PA2 cells (7PA2-CM), which contains naturally secreted Aβ oligomers [28], also reduced the synaptophysin content of cortical neurones, whereas conditioned medium from non-transfected Chinese hamster ovary cells (CHO-CM), which did not contain Aβ oligomers, had no affect (Figure 4A). Pre-treatment of cortical neurones with either 1 μM AACOCF_3 _or 1 μM MAFP prevented the 7PA2-CM mediated decrease in synaptophysin (Figure 4B).

### PLA_2 _inhibitors did not affect the accumulation of PrP82-146 in synapses

**Figure 5:**
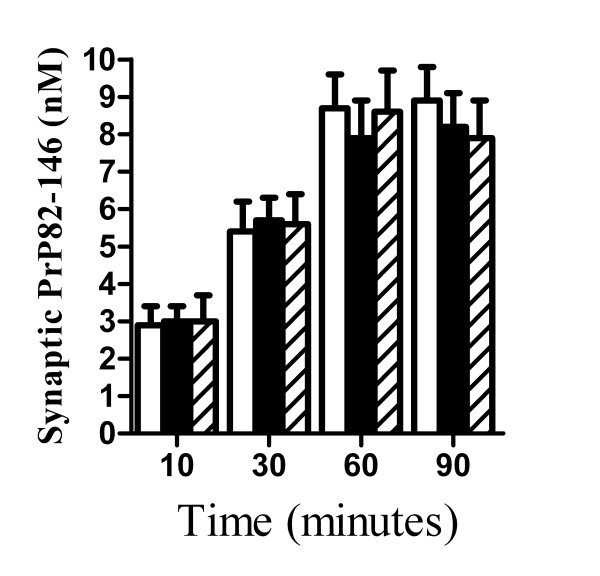
**PLA_2 _inhibitors did not affect the accumulation of PrP82-146 in synapses**. The amount of PrP82-146 detected in synaptosomes isolated from cortical neurones incubated with a vehicle control (■), 1 μM AACOCF_3 _(□) or 1 μM MAFP (striped bars). Treated cortical neurones were incubated with 100 nM PrP82-146 for the time periods shown. Synaptosomes were isolated from neuronal cultures and the amount of PrP82-146 was measured by ELISA. Values shown are the mean average amount of PrP82-146 (nM) ± SD, n = 9.

Prior studies showed that PrP82-146 accumulated within synaptosomes isolated from cortical neurones prior to synaptic degeneration [8]. That observation raised the possibility that the protective effect of PLA_2 _inhibitors might be because they reduced the binding/accumulation of PrP82-146 within synapses. Firstly, we showed that the amount of synaptophysin was not significantly different between synaptosomes isolated from cortical neurones and neurones treated 1 μM AACOCF_3 _or 1 μM MAFP; indicating that these drugs did not affect synapse formation. Next cortical neurones were pre-treated with 1 μM AACOCF_3 _before the addition of 100 nM PrP82-146. The amount of PrP82-146 found within synaptosomes isolated from vehicle and AACOCF_3 _treated neurones were not significantly different (Figure 5). Similar results were obtained with synaptosomes from cortical neurones pre-treated with 1 μM MAFP indicating that the PLA_2 _inhibitors did not prevent the binding or transport of PrP82-146 to synapses.

### PLA_2 _inhibitors protected against synapse degeneration induced by PLAP

**Figure 6:**
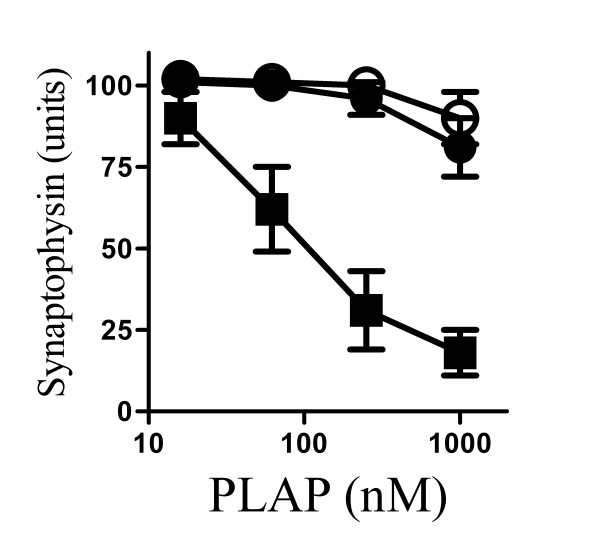
**PLA_2 _inhibitors protected against PLAP induced synapse degeneration**. The synaptophysin content of cortical neurones pre-treated with a vehicle control (■), 1 μM AACOCF_3 _(○) or 1 μM MAFP (●) and incubated with varying concentrations of PLAP for 24 hours. Values shown are the mean average amount of synaptophysin ± SD, n = 12.

Next we sought to determine whether synapse degeneration resulted from activation of an endogenous PLA_2_. The addition of PLAP, a peptide that activated endogenous cPLA_2 _[29,30], had similar effects as PrP82-146 and Aβ_1-42 _on synapses; it reduced the synaptophysin content of cortical neurones in a dose dependent manner. The addition of 1 μM PLAP reduced the synaptophysin content of cultured neurones by approximately 80% without affecting the survival of neurones as measured by thiazyl blue tetrazolium (96% neuronal survival ± 5 compared with 100% ± 8, n = 9, P = 0.7). Pre-treatment with 1 μM AACOCF_3 _or 1 μM MAFP protected cortical neurones against PLAP induced synapse degeneration; in vehicle treated neurones the EC_50 _of PLAP was 100 nM, while in neurones pre-treated with 1 μM AACOCF_3 _or 1 μM MAFP the addition of 1 μM PLAP did not reduce the synaptophysin content by more than 20% (Figure 6).

### cPLA_2 _is enriched in synaptosomes

**Figure 7:**
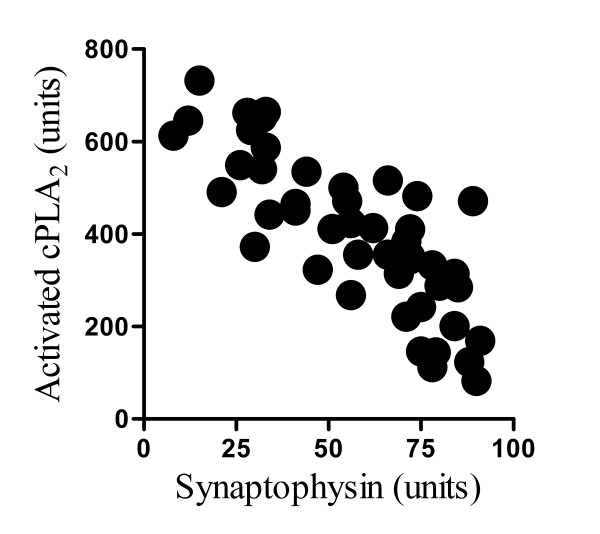
**PrP82-146 increased activation of cPLA_2 _in synapses**. Correlation between the amounts of activated cPLA_2 _in synaptosomes isolated from primary cortical neurones 1 hour after the addition of varying concentrations of PrP82-146 and the amount of synaptophysin in cell extracts from primary cortical neurones incubated for 24 hours with the same concentrations of PrP82-146.

To investigate the hypothesis that synapse degeneration resulted from activation of an endogenous cPLA_2_, the amount of cPLA_2 _protein in synaptosomes was measured. The cPLA_2 _protein was enriched in synaptosomes isolated from cortical neurones compared to whole cell membrane extracts (100 units cPLA_2_/mg protein ± 31 compared to 6 units/mg ± 1, n = 12, P < 0.01). There were no significant differences in the amount of total cPLA_2 _protein in synaptosomes isolated from vehicle treated cortical neurones and those treated for 1 hour with 200 nM PrP82-146, 200 nM PrP82-146scrambled, 1 μM PLAP, 100 nM Aβ_1-42 _or 100 nM Aβ_42-1 _(Table 1). Measurements were made after 1 hour, before any synapse degeneration was observed. Next specific antibodies (to cPLA_2 _phosphorylated at serine 505) were used to determine the amount of activated cPLA_2 _within synaptosomes. The amount of activated cPLA_2 _within synaptosomes was significantly increased following the addition of 200 nM PrP82-146, 1 μM PLAP or 100 nM Aβ_1-42 _but not after the addition of PrP82-146scrambled or Aβ_42-1 _(Table 1). Moreover, there was a significant inverse correlation between the amount of activated cPLA_2_in synaptosomes after 1 hour and the amount of synaptophysin in neurones after 24 hours following the addition of different amounts of PrP82-146 (range 1.25 to .005 μM), Pearson's coefficient = -0.76, P < 0.01 (Figure 7).

**Table 1:** PrP82-146 and Aβ_1-42_**activated cPLA**_2_**within synapses**.

Treatment	**Total cPLA**_2 _**protein** (units/mg protein)	**Activated cPLA**_2_ (units/mg protein)
**None (units)**	100 ± 31	100 ± 19

**200 nM PrP82-146**	106 ± 18	773 ± 51 *

**200 nM PrP82-146scrambled**	95 ± 22	111 ± 34

**1 μM PLAP**	119 ± 30	928 ± 84 *

**100 nM Aβ**_1-42_	109 ± 21	655 ± 62 *

**100 nM Aβ**_42-1_	92 ± 27	121 ± 34

Primary cortical neurones were incubated with PrP, Aβ or PLAP peptides as shown for 1 hour. Synaptosomes were subsequently isolated from treated neurones and the amount of cPLA_2 _protein, and the amount of activated cPLA_2 _(phosphorylated at serine 505) were measured by ELISA. Values shown are the mean average amount of cPLA_2 _protein or activated cPLA_2 _(units/mg protein) ± SD, n = 12. * = amount of activated cPLA_2 _significantly greater than in control synaptosomes.

### PAF antagonists protected against PrP82-146 induced synapse degeneration

**Figure 8:**
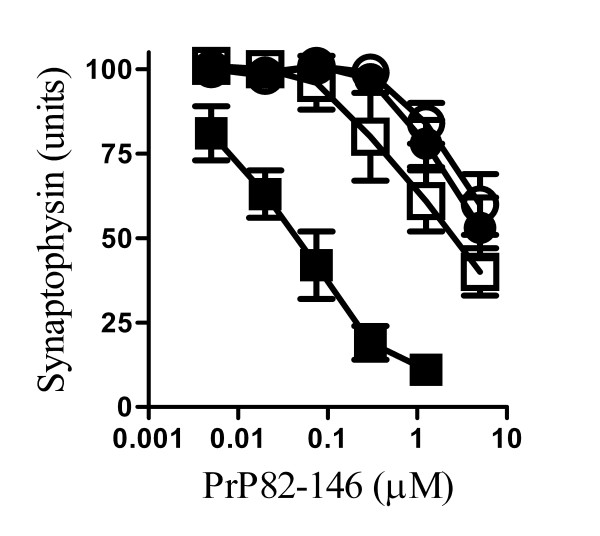
**PAF receptor antagonists protected against PrP82-146 induced synapse degeneration**. The synaptophysin content of cortical neurones pre-treated with a vehicle control (■), 1 μM ginkgolide B (○), 2 μM Hexa-PAF (●) or 2 μM CV6209 (□) and subsequently incubated with varying concentrations of PrP82-146 for 24 hours. Values shown are the mean average amount of synaptophysin ± SD, n = 12.

The activation of PLA_2 _is the first step in the production of PAF [31] that has been shown to cause synapse degeneration *in vitro *[27]. The addition of PAF receptor antagonists (Hexa-PAF, CV6209 or ginkgolide B), in the range 0.1-10 μM, did not affect the amount of synaptophysin in cortical neurones. However, pre-treatment with 2 μM Hexa-PAF, 2 μM CV6209 or 1 μM ginkgolide B provided protection against PrP82-146 induced synapse degeneration (Figure 8). The EC_50 _of PrP82-146 in vehicle treated cortical neurones was 50 nM while the EC_50 _of PrP82-146 for neurones treated with 2 μM Hexa-PAF was 2 μM, for neurones treated with 2 μM CV6209 the EC_50 _was 5 μM and for neurones pre-treated with 1 μM ginkgolide B the EC_50 _was 20 μM. Pre-treatment with Hexa-PAF, ginkgolide or CV6209 also prevented the reduction in the synaptophysin content of cortical neurones incubated with 100 nM Aβ_1-42 _or 1 μM PLAP (Table 2). In a similar manner, the amount of synaptophysin in hippocampal neurones incubated with 50 nM PrP82-146 was significantly increased by pre-treatment with 1 μM ginkgolide B (97 units synaptophysin ± 9 compared to 18 ± 7, n = 6, P < 0.01), 2 μM Hexa-PAF (94 ± 11 compared to 18 ± 7, n = 6, P < 0.01) or 2 μM CV6209 (94 ± 11 compared to 18 ± 7, n = 6, P < 0.01).

**Table 2:** PAF receptor antagonists reduced synapse degeneration induced by Aβ_1-42_**or PLAP**.

	**Synaptophysin (units/10**^**6**^**cells)**			
**Treatment**	**Vehicle**	**Hexa-PAF**	**Ginkgolide B**	**CV6209**

**Vehicle**	100 ± 6	98 ± 7	102 ± 5	96 ± 8

**100 nM Aβ**_1-42_	32 ± 6	94 ± 6	98 ± 9	89 ± 8

**1 μM PLAP**	27 ± 7	95 ± 8	94 ± 8	82 ± 7

Primary cortical neurones were pre-treated with a vehicle control, or with PAF receptor antagonists (2 μM Hexa-PAF, 1 μM ginkgolide or 2 μM CV6209) for 1 hour prior to incubation with Aβ_1-42 _or PLAP as shown for 24 hours. Values shown are the mean average amount of synaptophysin (units/10^6 ^cells) ± SD, n = 12.

### Prostaglandin E_2 _induced synapse degeneration

**Figure 9:**
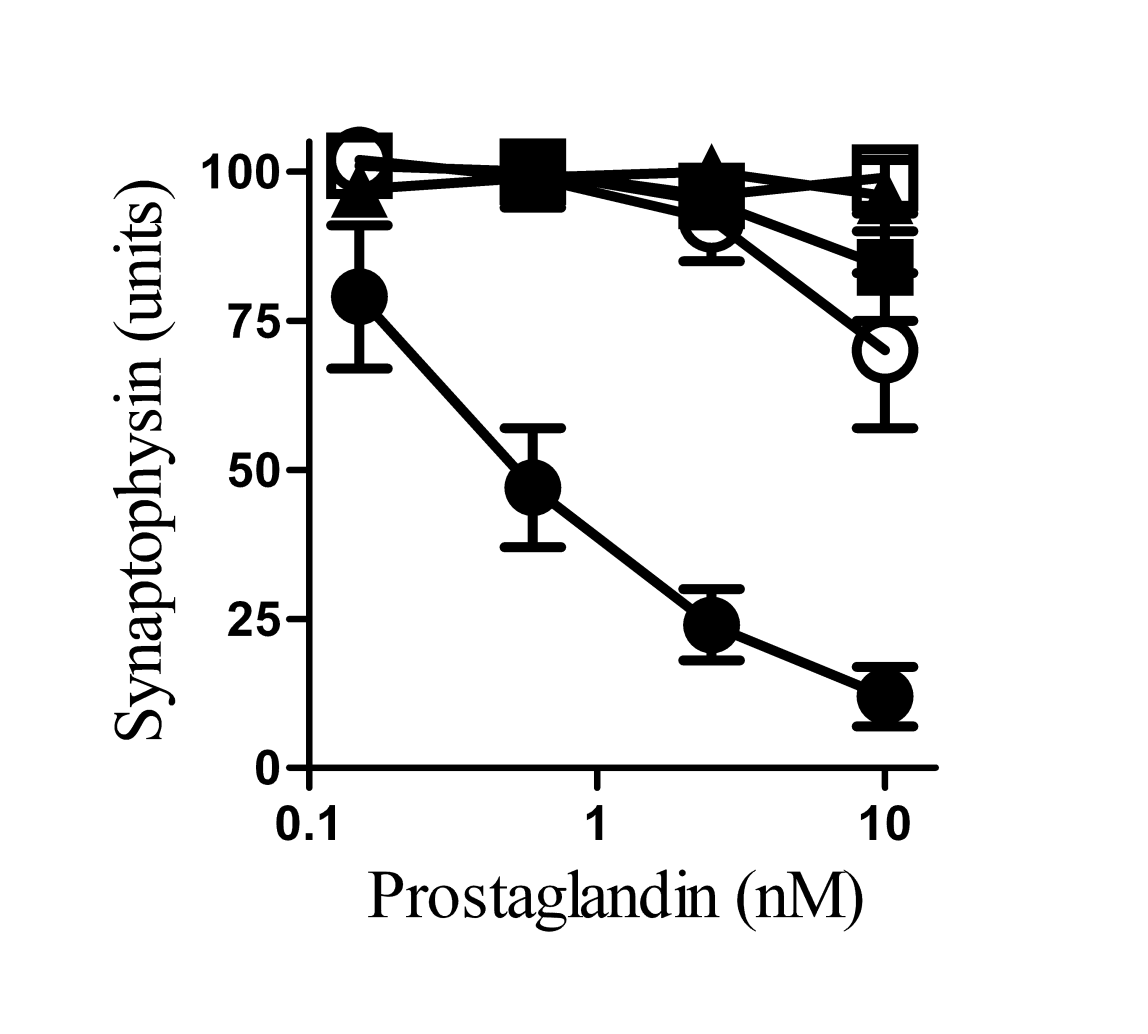
**Prostaglandin E_2 _caused synapse degeneration**. The synaptophysin content of cortical neurones treated with varying concentrations of prostaglandins E_2 _(●) D_2 _(○), F_2α_(□), I_2 _(■) or 15d-J_2 _(▲) for 24 hours. Values shown are the mean average amount of synaptophysin ± SD, n = 9.

Prior studies showed that PAF facilitates the production of prostaglandins [32] suggesting that one or more of the prostaglandins produced in response to PrP82-146, Aβ_1-42 _or PLAP are responsible for synapse degeneration. This hypothesis was tested by treating cortical neurones with individual prostaglandins. We report that the addition of prostaglandin E_2_, but not other prostaglandins reduced the amount of synaptophysin in cortical neurones (Figure 9). Prostaglandin E_2_, acts via specific prostanoid E receptors [33] and pre-treatment with the prostanoid E receptor antagonist AH13205, but not the prostanoid D receptor antagonist BWA868C, prevented the loss of synaptophysin in cortical neurones incubated with PrP82-146, Aβ_1-42_, PLAP, PAF or prostaglandin E_2 _(Table 3). Taken together, these results show that the effects of PrP82-146 or Aβ_1-42 _on synapses were ultimately mediated through prostanoid E receptors.

**Table 3:** A prostanoid E receptor antagonist protected against synapse degeneration

	Synaptophysin (units)		
**Treatment**	**Vehicle control**	**Prostanoid E receptor antagonist AH13205**	**Prostanoid D receptor antagonist BWA868C**

**Vehicle control**	100 ± 6	99 ± 7	99 ± 3

**200 nM PrP82-146**	22 ± 5	87 ± 8	30 ± 7

**100 nM Aβ**_**1-42**_	32 ± 6	93 ± 5	34 ± 6

**1 μM PLAP**	27 ± 7	91 ± 7	38 ± 6

**10 nM PAF**	31 ± 4	85 ± 9	32 ± 5

**10 nM Prostaglandin E**_**2**_	30 ± 5	97 ± 3	33 ± 6

Primary cortical neurones were pre-treated with a vehicle control, a prostanoid E receptor antagonist (100 nM AH13205) or a prostanoid D receptor antagonist (100 nM BWA868C) for 1 hour prior to incubation with PrP82-146, Aβ_1-42_, PLAP, PAF or prostaglandin E_2 _as shown for 24 hours. Values shown are the mean average amount of synaptophysin ± SD, n = 9.

## Discussion

The addition of PrP82-146 reduced the synaptophysin content of cortical neurones indicative of a loss of synapses. This occurred at concentrations of peptide that did not affect neuronal survival [8], results consistent with *in vivo *observations that the loss of synapses in prion diseases occurs before any significant neuronal death is seen [1,2]. PrP82-146 also damaged synapses in hippocampal neuronal cultures. The hippocampus is involved in memory formation and the loss of synapses in this area is consistent with the cognitive deficits that occur during the early stages of scrapie infections [3,34].

A pharmacological approach was used to examine the molecular mechanisms involved in prion induced synapse degeneration. Critically, pre-treatment with selective PLA_2 _inhibitors reduced PrP82-146 induced synapse degeneration, whereas pre-treatment with inhibitors of some other common cell signalling pathways including phospholipase C had no affect. PrP^Sc ^is detected within synapses in CJD patients and experimental scrapie infections [2,35,36] and PrP82-146 was found within synaptosomes isolated from cultured neurones [8]. We found that pre-treatment with PLA_2 _inhibitors did not alter the amount of PrP82-146 found within synaptosomes, indicating the protective effect of these drugs was not due to the reduced binding of PrP82-146 to synaptic receptors, or a reduction in the trafficking of PrP82-146 to synapses.

Next, the possibility that PrP82-146 might activate cPLA_2 _resident within synapses was examined. Previous studies reported that PLA_2 _was found at synapse and regulated synaptic vesicle recycling [37,38] and we observed that cPLA_2 _was highly enriched in synaptosomes. These observations imply that cPLA_2 _is part of the normal function of synapses and complete inhibition of cPLA_2 _may not be desirable as it may interfere with neurotransmission. However, the addition of PrP82-146 increased the amount of activated cPLA_2 _within synaptosomes nearly 8 fold suggesting that PrP82-146 induced activation of cPLA_2 _resident within synapses was responsible for synapse degeneration. This hypothesis was supported by our observations that there was a significant inverse correlation between the amount of activated cPLA_2 _and synaptic density and that synapse degeneration was also observed after the addition of PLAP. In addition, the effect of both PLAP and PrP82-146 on synapses was greatly reduced by pre-treatment with the cPLA_2 _inhibitors AACOCF_3 _and MAFP. Collectively, these results strongly suggesting that activation of an endogenous cPLA_2 _is a pivotal event in PrP82-146 mediated synapse degeneration.

The activation of PLA_2 _leads to the formation of a number of bioactive factors including prostaglandins, leucotrienes, docosanoids and PAF [31,39]. PAF receptors are present at synaptic endings [40] and PAF facilitates neurotransmission [41]. However, higher concentrations of PAF are implicated in the neurotoxicity of glutamate, human immunodeficiency virus infection, prion diseases and AD [39,42]. Reports that synaptic activity becomes excitotoxic in neurones exposed to elevated levels of PAF [43] may explain the role of PAF in these diseases. Here we showed that synapse degeneration induced by PrP82-146 was reduced by pre-treatment with PAF receptor antagonists, ginkgolide B, Hexa-PAF and CV6209. These PAF receptor antagonists also reduced synapse degeneration induced by PLAP suggesting that the effect of PrP82-146 and PLAP were a secondary effect mediated by activation of an endogenous cPLA_2 _and the production of PAF.

PAF affects the production of prostaglandin E_2 _[32] which is found in close association with PrP^Sc ^deposition and neuronal degeneration in scrapie [44]. In addition, the levels of prostaglandin E_2_are raised in the cerebrospinal fluid of patients with CJD [45]. While prostaglandin E_2 _may modify hippocampal synaptic transmission via a pre-synaptic prostanoid E receptors [46], higher concentrations cause synapse degeneration [27]. As shown in Figure 9 the addition of prostaglandin E_2_, but not other prostaglandins, caused synapse degeneration. The effects of prostaglandin E_2 _are mediated by specific membrane bound prostanoid E receptors [47] and synapse degeneration induced by PrP82-146 was reduced by pre-treatment with the prostanoid E receptor antagonist AH13205 suggesting that its effect was mediated through the prostanoid E receptor.

The results presented here may also be pertinent for other diseases in which synapse degeneration is common. For example, the neurotoxicity of Aβ_1-42_, widely believed to be the main toxin responsible for neurodegeneration in AD [23,48-50], was reduced following knockdown on cPLA_2 _[51,52]. In this study we showed that Aβ_1-42 _strongly activated cPLA_2 _within synapses and that pre-treatment with PLA_2 _inhibitors protected against the synapse degeneration induced by Aβ_1-42_and naturally secreted Aβ oligomers (7PA2-CM). This observation is consistent with reports that inhibition of cPLA_2 _protected against cognitive impairment in a mouse model of AD [53]. A recent study indicated that PrP^C ^acts as a receptor for Aβ_1-42 _oligomers [54]. PrP^C ^also acts as a receptor for PrP peptides [55] suggesting that both PrP and Aβ_1-42 _activate PLA_2 _through their interaction with PrP^C^. We note that oligomers, rather than monomers, of PrP or Aβ_1-42 _are neurotoxic [56-58]. Since oligomers of PrP and Aβ_1-42_, but not monomers, have the capacity to cross-link PrP^C^, our results support the hypothesis that it is the cross-linkage of PrP^C ^by PrP or Aβ_1-42 _oligomers that activated cPLA_2 _leading to the production of PAF and prostaglandins. We propose that it is the persistent activation of cPLA_2 _by PrP82-146 or Aβ_1-42 _that results in high concentrations of PAF and prostaglandin E_2 _that cause synapse degeneration [27,43].

## Conclusion

Drugs that protect the synapse provide a rational strategy to treat many neurodegenerative diseases. In this study we showed that PLA_2 _inhibitors, PAF receptor antagonists and a prostanoid E receptor antagonist all protected against the synapse degeneration induced by PrP82-146 or Aβ_1-42 _*in vitro*. These peptides did not have a direct effect on synapses; rather synapse degeneration was caused after PrP82-146 or Aβ_1-42 _induced activation of an endogenous cPLA_2 _and the production of PAF and prostaglandin E_2_. Such results suggest that specific cPLA_2 _inhibitors or PAF receptor antagonists that are able to cross the blood-brain barrier should be considered for further testing in animal models of prion and Alzheimer's diseases.

## Methods

### Primary neuronal cultures

Primary cortical neurones were prepared from the brains of mouse embryos (day 15.5). Neuronal precursors were plated at 2 × 10^5 ^cells/well in 48 well plates in Ham's F12 containing 5% foetal calf serum (FCS) for 2 hours. Cultures were shaken (600 r.p.m for 5 minutes) and non-adherent cells removed by 3 washes in phosphate buffered saline (PBS). Neurones were grown in neurobasal medium (NBM) containing B27 components (PAA) for 7 days. Immunohistochemistry showed that the cells were greater than 97% neurofilament positive. Less than 3% stained for GFAP (astrocytes) or for F4/80 (microglial cells). Hippocampal neurones were prepared from the brains of adult mice as described [59]. Briefly, hippocampi were dissected and triturated in Ham's F12 containing 5% FCS, 0.35% glucose, 0.025% trypsin, and 0.1% type IV collagenase. After 30 minutes at 37°C, the cells were triturated again and the cell suspension was passed through a 100 μM cell strainer. Cells were collected by centrifugation, washed twice in Ham's F12 containing 5% FCS and plated at 2 × 10^5 ^cells/well in 48 well plates for 24 hours. Cultures were shaken (600 r.p.m for 5 minutes) to remove non-adherent cells, washed twice with PBS and neurones were cultured in NBM containing B27 components and 10 ng/ml glial-derived neurotrophic factor (Sigma) for 10 days. Neurones were incubated with test compounds for 3 hours before the addition of peptides and the amount of synaptophysin in treated neurones was measured 24 hours later. The survival of neurones was determined 5 days later using 25 μM thiazlyl blue tetrazolium for 3 hours; neuronal survival was reported as a percentage of controls (vehicle treated neurones).

### Cell extracts

Neurones were homogenised in a buffer containing 150 mM NaCl, 10 mM Tris-HCl, 10 mM EDTA, 0.5% Nonidet P-40, 0.5% sodium deoxycholate and mixed protease inhibitors (AEBSF, Aprotinin, Leupeptin, Bestain, Pepstatin A and E-46 (Sigma)) and a phosphatase inhibitor cocktail including PP1, PP2A, microcystin LR, cantharidin and p-bromotetramisole (Sigma) at 10^6 ^cells/ml. Nuclei and cell debris was removed by low speed centrifugation (300 × *g *for 5 minutes). For immunoblots, cells were mixed 1:1 with Laemmli buffer containing β-mercaptoethanol, boiled for 5 minutes and run on a 12% polyacrylamide gel. Proteins were transferred onto a Hybond-P PVDF membrane (Amersham Biotech) by semi-dry blotting. Membranes were blocked using 10% milk powder; synaptophysin was detected using a mouse monoclonal antibody (mAb) anti-synaptophysin SY38 (Abcam) and β-actin was detected by incubation with a mouse mAb (clone AC-74, Sigma). These were visualised using a combination of biotinylated rabbit anti-mouse IgG (Dako), extravidin-peroxidase and an enhanced chemiluminescence kit (Invitrogen).

### Synaptophysin ELISA

Synaptophysin levels in cell extracts were measured by ELISA as described [9,27]. A mouse monoclonal anti-synaptophysin MAB368 (Millipore) was used as a capture antibody and bound synaptophysin was detected using rabbit polyclonal anti-synaptophysin (Abcam) followed by a biotinylated anti-rabbit IgG, extravidin-alkaline phosphatase and 1 mg/ml 4-nitrophenol phosphate (Sigma). Absorbance was measured on a microplate reader at 405 nm and synaptophysin content was calculated from a standard curve. Samples were expressed as "units synaptophysin" where 100 units was defined as the amount of synaptophysin in 10^6 ^vehicle treated neurones. A standard curve was generated from this sample using sequential log 2 dilutions (range 100 to 1.56 units).

### Synaptosome preparations

Cortical neurones were pre-treated with drugs for 3 hours prior to the addition of 100 nM PrP82-146. After different time points (10, 30, 60 or 90 minutes) neurones were washed 3 times to remove unbound peptide and synaptosomes prepared on a discontinuous Percoll gradient as described [60]. Briefly, 10^6 ^cortical neurones were homogenized at 4°C in 1 ml of SED solution (0.32 M sucrose, 50 mM Tris-HCl, 1 mM EDTA, and 1 mM dithiothreitol, pH 7.4 and mixed protease/phosphates inhibitors (as above)). The preparation was centrifuged at 1000 × *g *for 10 minutes. The supernatant was transferred to a 4-step gradient of 3, 7, 15, and 23% Percoll in SED solution and centrifuged at 16,000 × *g *for 30 minutes at 4°C. The synaptosome fraction was collected from the interface of the 15% and 23% Percoll steps, washed twice (16,000 × *g *for 30 minutes at 4°C) and suspended in extraction buffer (150 mM NaCl, 10 mM Tris-HCl, 10 mM EDTA, 0.2% SDS and mixed protease/phosphatase inhibitors).

### PrP82-146 ELISA

The amount of PrP82-146 in synaptosomes was determined by ELISA. Nunc Maxisorb Immunoplates were coated with 0.5 μg/ml of mouse mAb 3F4 (reactive with residues 109-112 of human PrP (Abcam)) this mAb does not bind to murine PrP [61]. Samples were applied and PrP82-146 was detected with 0.5 μg/ml biotinylated ICSM35 (D-gen, http://www.d-gen.co.uk), followed by streptavidin-alkaline phosphatase (Dako) and 1 mg/ml 4-nitrophenyl phosphate (Sigma). Absorbance was measured on a microplate reader at 405 nm and the amount of PrP82-146 in cell extracts was calculated by reference to a standard curve of PrP82-146.

### cPLA_2 _ELISA

The amount of cPLA_2 _in synaptosomes was measured by ELISA [62]. Nunc Maxisorb Immunoplates were coated with 0.5 μg/ml of mouse mAb anti-cPLA_2_, clone CH-7 (Upstate) and blocked with 10% FCS. Samples were incubated for 1 hour and the amount of cPLA_2 _was detected using a goat polyclonal anti-cPLA_2 _(Santa-Cruz Biotech). Bound antibodies were detected with biotinylated anti-goat IgG, extravidin-alkaline phosphatase and 1 mg/ml 4-nitrophenyl phosphate. Absorbance was measured at 405 nm and the amount of cPLA_2 _was calculated from a standard curve using nonlinear regression. The amount of cPLA_2 _protein was expressed in units (100 units = amount of cPLA_2 _in synaptosomes isolated from 10^6 ^untreated primary cortical neurones). The activation of cPLA_2 _is accompanied by phosphorylation of the 505 serine residue which creates a unique epitope. To measure the amount of activated cPLA_2_, an ELISA using a mAb (anti-cPLA_2_, clone CH-7) combined with rabbit polyclonal anti-phospho-cPLA_2 _(Cell Signalling Technology) was used. Bound antibodies were detected with biotinylated anti-rabbit IgG (Dako), extravidin-alkaline phosphatase and 1 mg/ml 4-nitrophenyl phosphate. Samples were expressed as "units activated cPLA_2_" where 100 units were defined as the amount of activated cPLA_2 _in synaptosomes derived from 10^6 ^untreated primary cortical neurones.

### Drugs

U73122 and Ethyl-18-OCH_3 _were obtained from Biomol. Arachidonyl trifluoromethyl ketone (AACOCF_3_), aristolochic acids, ginkgolide B, 1-O-Hexadecyl-2-acetyl-*sn*-glycerol-3-phospho-(N,N,N-trimethyl)-hexanolamine (Hexa-PAF), methyl arachidonyl fluorophosphonate (MAFP), prostaglandin G_2_, AH13205 and BWA868C were obtained from Sigma. Prostaglandins 15d-J_2_, E_2_, D_2_, F_2_α, H_2 _and I_2 _were obtained from Novabiochem. Stock solutions were dissolved in ethanol or di-methyl sulphoxide (DMSO) and diluted in medium to obtain final working concentrations. Vehicle controls consisted of equivalent dilutions of ethanol or DMSO in NBM.

### Peptides

Peptides containing amino acids 82 to 146 of the human PrP protein (PrP82-146) corresponding to a PrP fragment found in certain prion-infected human brains [7], a control peptide (PrP82-146scrambled) were synthesised by solid-phase chemistry and purified by reverse-phase high performance liquid chromatography. A synthetic peptide containing the amino acid residues 1 to 42 of Aβ (Aβ_1-42_) of the Aβ protein and a control peptide consisting of the same amino acids in reverse order (Aβ_42-1_) were obtained from Bachem. Aβ peptides were first dissolved in hexafluoroisopropanol, lyophilised and subsequently solubilised and stored in DMSO. PLA_2 _Activating Peptide (PLAP) was purchased from Bachem. Stock solutions of peptides were thawed on the day of use and sonicated before dilution in culture medium and addition to cells.

CHO cells stably transfected with a cDNA encoding APP_751 _containing the Val717Phe familial AD mutation (referred to as 7PA2 cells) were cultured in DMEM with 10% FCS [28]. Conditioned medium (CM) from these cells contains stable Aβ oligomers (7PA2-CM). Conditioned medium from non-transfected CHO cells (CHO-CM) was used as controls.

### Statistical Methods

Differences between treatment groups were assessed using two sample t tests or analysis of variance techniques. In all tests statistical significance was set at the 1% level.

## List of abbreviations

(AD): Alzheimer's disease; (Aβ): amyloid-β; (AACOCF_3_): Arachidonyl trifluoromethyl ketone; (CHO): Chinese hamster ovary; (CM): conditioned medium; (CJD): Creutzfeldt-Jakob disease; (DMSO): di-methyl sulphoxide; (ELISA): Enzyme linked immunoassay; (Hexa-PAF): 1-O-Hexadecyl-2-acetyl-*sn*-glycerol-3-phospho-(N,N,N-trimethyl)-hexanolamine; (MAFP): methyl arachidonyl fluorophosphonate; (NBM): neurobasal medium; (PBS): phosphate buffered saline; (PLA_2_): phospholipase A_2_; (cPLA_2_): cytoplasmic PLA_2_; (PLAP): PLA_2 _Activating Peptide; (PAF): platelet-activating factor; (PrP): prion protein.

## Competing interests

The authors declare that they have no competing interests.

## Authors' contributions

CB and MT were responsible for the conception, planning and performance of experiments and for writing the manuscript. AW contributed to the planning of experiments, interpretation of results and the writing of the manuscript. All authors approved the final manuscript.
